# Neuropsychological functioning, age, and medication adherence in bipolar disorder

**DOI:** 10.1371/journal.pone.0184313

**Published:** 2017-09-05

**Authors:** Nadia Corréard, Julia-Lou Consoloni, Aurélie Raust, Bruno Etain, Romain Guillot, Sophie Job, Joséphine Loftus, Isabelle Médecin, Thierry Bougerol, Mircea Polosan, Benjamin Fredembach, Sébastien Gard, Katia M’Bailara, Jean-Pierre Kahn, Paul Roux, Anne-Sophie Homassel, Mathilde Carminati, Lucile Matos, Emilie Olié, Frank Bellivier, Philippe Courtet, Chantal Henry, Marion Leboyer, Jean-Michel Azorin, Raoul Belzeaux, FACE-BD collaborators

**Affiliations:** 1 Department of Psychiatry, AP-HM, Marseille, France; 2 FondaMental foundation, Foundation of scientific cooperation, Créteil, France; 3 CRN2M-UMR7286, Aix-Marseille University, CNRS, Marseille, France; 4 AP-HP, Academic Hospital Henri Mondor, Psychiatric and Addictology pole, Créteil, France; 5 AP-HP, Fernand Widal Hospital, Department of Addictology-Toxicology-Psychiatry and University Paris-7, Paris, France; 6 Department of Psychiatry and Clinical Psychology, Psychotherapeutic Centre of Nancy, Laxou, France; 7 Department of Psychiatry, Princess-Grace Hospital, Monaco, Monaco; 8 Department of Psychiatry, Academic Hospital of Grenoble, Grenoble, France; 9 U1216 INSERM-UGA – Brain stimulation and Systems neuroscience, Grenoble Institute of Neurosciences, La Tronche, France; 10 Charles-Perrens Hospital, Department of clinical and academic Psychiatry, Bordeaux, France; 11 University of Bordeaux, Laboratory of psychology, Bordeaux, France; 12 French Addictovigilance network (CEIP-A) CHRU of Nancy, Nancy, France; 13 University of Lorraine, Nancy, France; 14 Department of Psychiatry for adults, Academic Hospital of Versailles, UFR of Health Sciences Simone Veil, University of Versailles Saint-Quentin en Yvelines, Versailles, France; 15 CHRU Lapeyronie, Department of Emergency Psychiatry and Post-Acute Care, Montpellier, France; 16 Inserm, U1061, University of Montpellier, Montpellier, France; 17 Inserm, U955, Translational Psychiatry, Mondor Institute, Créteil, France; 18 CNRS, UMR 7289, Institute of Neurosciences Timone, Marseille, France; RIKEN Brain Science Institution, JAPAN

## Abstract

**Objectives:**

Poor adherence to medication is frequent in bipolar disorder (BD) and has been associated with several factors. To date, the relationship between low adherence and neuropsychological functioning in BD is still unclear. As age and neuropsychological functioning might have opposing influences on adherence, our aim was to investigate this link with a particular focus on the effect of age.

**Methods:**

In a cross-sectional study, we included 353 patients divided into two age-groups (16–46; 47–71) from a French cohort diagnosed with BD (type I, II, NOS) and strictly euthymic. All patients had a standardized clinical and neuropsychological assessment and were categorized as high (n = 186) or low (n = 167) adherent based on their score from the Medication Adherence Rating Scale. Clinical information was collected based on a standardized interview and clinical validated scales. Neuropsychological performances were evaluated with an established standardized neuropsychological battery for bipolar disorder patients. After univariate analysis, neuropsychological and clinical predictors of low adherence were included in two age-specific stepwise multiple logistic regressions.

**Results:**

A smaller number of hospitalizations (OR = 0.846, p = 0.012), a shorter illness duration (OR = 0.937, p = 0.003) and higher adverse effects (OR = 1.082, p<0.001) were associated with a greater risk of low adherence in the younger patients. In the older patients, low adherence was also predicted by a smaller number of hospitalizations (OR = 0.727, p = 0.008) and higher adverse effects (OR = 1.124, p = 0.005). Interestingly poor inhibition performance was also a significant predictor of low adherence in older patients (OR = 0.924, p = 0.030).

**Conclusions:**

We found an age-specific relationship between cognitive functioning and adherence in patients with BD. Poor inhibition performances predicted low adherence in older patients only. Our results highlight the need to provide age-adapted therapeutic interventions to improve adherence in patients with BD.

## Introduction

Bipolar disorder (BD) is a chronic and severe mental disorder often characterized by residual symptoms as well as heterogeneous impairment of cognitive functioning [1–3]. Pharmacological treatment is essential to treat symptomatic mood episodes and to prevent relapses and recurrences [4]. Unfortunately, treatment nonadherence is frequent in BD. About 20% to 60% of patients are considered as poor or nonadherent without regard to the phase of the illness, including symptomatic remission periods [5, 6]. Treatment nonadherence has severe consequences. It is associated with more relapses, recurrences and an increased risk of suicide [7, 8]. Several factors have been related to low treatment adherence in BD [5, 6, 9]. We and others have demonstrated that male gender, depressive residual symptoms, and a higher level of medication side effects were associated with treatment nonadherence. Comorbidity such as substance use disorder has also been strongly associated with low treatment adherence in BD [10, 11]. Moreover, an increase in age of patients was linked with increased adherence to medication [9, 12, 13].

Otherwise, non-adherence to medication in chronic illnesses has been divided into two categories by researchers according to the patient’s perspective [14, 15]. Intentional non-adherence is defined as an active process whereby the patients voluntarily do not take the prescribed medication (i.e. Stopping or not taking medication or deciding to reduce the posology without informing the doctor) whereas unintentional non-adherence refers to unplanned and unconscious behaviors resulting to non-adherence. Unintentional non-adherence depends on the patients’ ability, and factors beyond their control, to follow the medical recommendations especially due to cognitive impairments (i.e. forgetting to take the medication). Therefore, researchers have started to investigate the association between cognitive impairments and medication adherence. An abundant literature has highlighted a link between neurocognitive dysfunction and treatment adherence in different diseases such as Parkinson disease, lupus erythematosus, and HIV infection [16–18]. Three previous studies have also suggested such an association in BD [19–21], while another study did not find any relationship between adherence and neurocognition [22]. Martinez-Aran et al [19] found that euthymic BD patients with poor adherence showed verbal learning and memory impairments as well as executive impairments in comparison to high adherent patients and healthy controls. As a consequence, to date, this relationship remains poorly studied and the results are still unclear.

Interestingly, on the one hand, age is associated with a decrease of neuropsychological performance across the lifespan of a healthy adult as well as in patients with BD [23–25]. Furthermore, aging worsens the cognitive impairments observed in BD [26]. On the other hand, increasing age is associated with better treatment adherence. Thus, age could be a major confounding factor when analyzing the relationship between adherence and neuropsychological functioning in patients. However, only one previous study examined the hypothesis of an age effect in the relationship between neurocognition and medication adherence, and this concerned patients with HIV infection [27]. In this study, neurocognitive impairment was associated with poorer medication adherence among older participants only. To the best of our knowledge, no previous study has been performed focusing on BD or other psychiatric disorders. A better understanding of the effect of cognitive impairments in BD patients is needed to develop and adapt techniques to improve adherence to medication in these patients and improve their quality of life.

We therefore aimed to explore the relationship between low treatment adherence and neuropsychological functioning of bipolar patients with a particular focus on the effect of age. We hypothesized that low treatment adherence would be associated with worse neuropsychological functioning in older patients.

## Materials and methods

### Study design and sample

**Fig 1:**
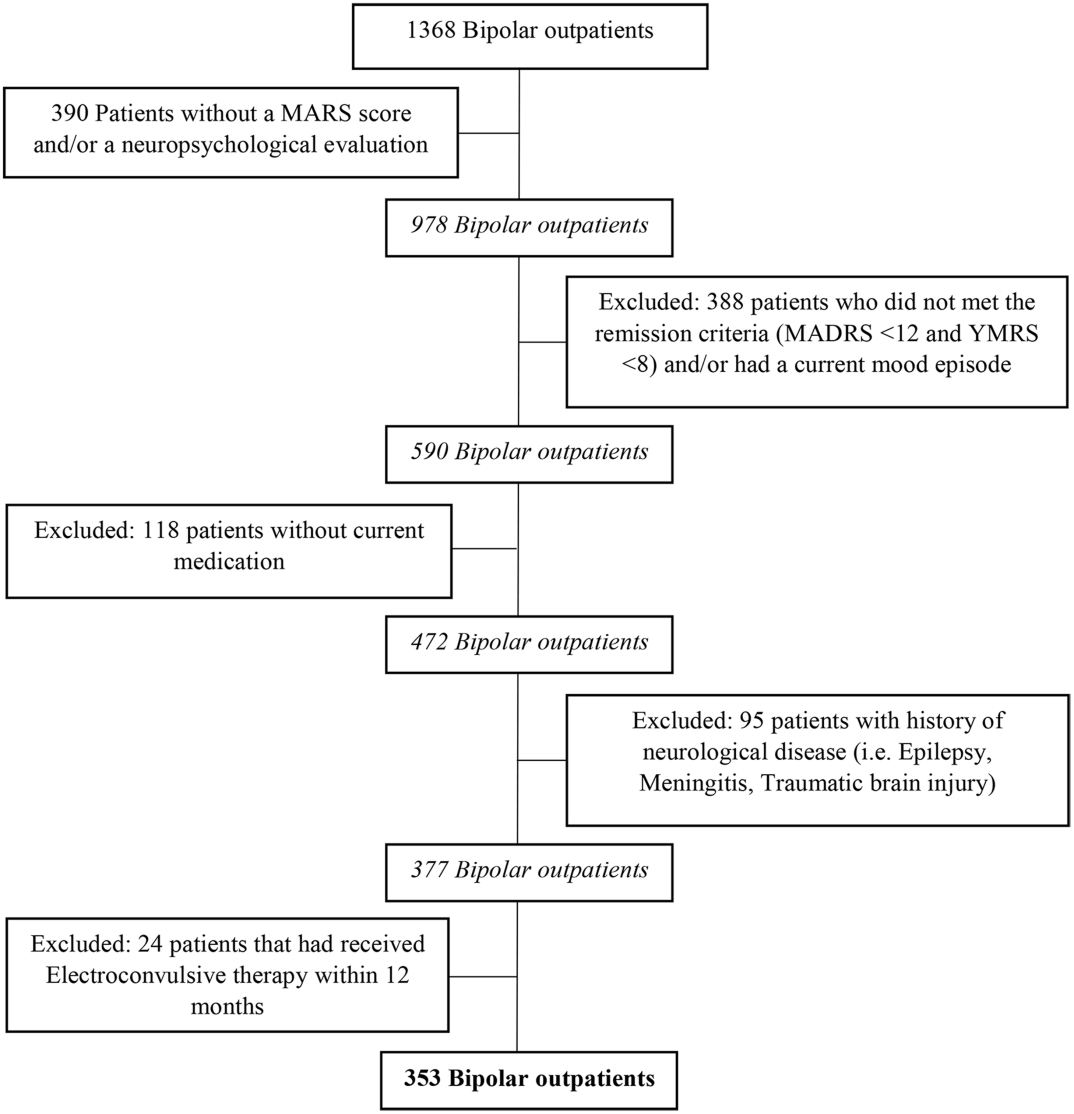
Selection procedure. Selection procedure of the final sample of bipolar outpatients (N = 353) from the FondaMental Advanced Centers of Expertise in Bipolar Disorders (FACE-BD) cohort.

We conducted a cross-sectional multicenter study involving the 9 French Expert Centers of the FondaMental foundation. We used data extracted from the FondaMental Advanced Centers of Expertise in Bipolar Disorders (FACE-BD) cohort [28]. Among the 1368 outpatients evaluated in the French FACE-BD from January 2009 to January 2015, we included 353 patients in this study diagnosed with BD (type I, II or Not Otherwise Specified (NOS)) according to the selection procedure described in Fig 1. Because of the worsening of cognitive impairment in patients with BD during the acute phase [1], only patients without a current mood episode for at least 3 months, according to the Diagnostic and Statistical Manual of Mental Disorders, 4th ed., revised (DSM-IV-TR) criteria [29] were included. Patients were included if they were in remission in accordance with the definition proposed by ISBD task force [30]; that is patients who scored <12 on the Montgomery-Asberg Depression Rating Scale (MADRS) [31] and <8 on the Young Mania Rating Scale (YMRS) [32] to avoid the confounding effects of mood symptoms. Since our aim was to clarify the relationship between neuropsychological functioning and medication adherence, we excluded patients without current specific pharmacological treatment. Patients with a history of neurological disease and patients who had received electroconvulsive therapy within 12 months were also excluded from the sample. As reported in previous studies, these two factors could affect neuropsychological functioning [33–36].

The assessment protocol was approved by the ethical review board (CPP-Ile de France IX, January, 18th; 2010). The ethical board requested that each patient receive an information letter. In this case, although written formal consent was not required, seeking permission from patients was a prerequisite to any analysis of the clinical data. A web-based application was developed to collate assessment data for clinical monitoring and research purposes. Access to the system was carefully regulated, and approval was obtained from the committee in charge of the safety of computerized databases (CNIL; DR-2011-069).

### Clinical assessment

The Structured Clinical Interview for DSM-IV-TR APA/2000 (SCID-I) [37] was used to determine diagnosis of BD I, II or NOS and all psychiatric comorbidities. Demographic and clinical variables were collected from the patient sample, including age, gender, level of education, lifetime psychotic symptoms, lifetime substance use disorder or smoking, lifetime anxiety disorder, number of hospitalizations, illness duration, and number and type of medication. Mood symptoms were evaluated through the MADRS and YMRS. Anxiety was evaluated by the State-Trait Anxiety Inventory [38]. Side effects were evaluated with the Patient Rated Inventory of Side Effects (PRISE)[39]. Treatment adherence was measured by the Medication Adherence Rating Scale (MARS) [40]. This scale consisted of a self-reporting instrument with 10 yes/no items (i.e. “Do you ever forget to take your medication?” or “It is unnatural for my mind and body to be controlled by medication”) [40–42]. The total score is obtained by summing the items. Patients with a score < 8 were categorized as *low adherent* since the low score was correlated with a low likelihood of medication adherence [40, 43] and patients with a total score ≥ 8 were qualified as *high adherent* since it was associated with a high likelihood of medication adherence [40, 43].

### Neuropsychological assessment

Trained professionals administered a 3-hour standardized cognitive battery that included some subtests of the Wechsler Adult Intelligence Scale (WAIS) [44, 45] and other neuropsychological tests. In fact, the neuropsychological tests and subtests used in our study are part of the BANC (Battery for Assessment of Neurocognition) established by the International Society for Bipolar Disorders in order to study cognitive impairments in bipolar disorder patients [46]. Because a new version of the battery (WAIS IV) was launched during the inclusion period, we used the percent of correct responses when the total number of items differed between the 2 versions. The neuropsychological battery assessed 5 cognitive domains:

- Intellectual functioning was assessed with percent of correct responses on the Vocabulary Subtest (asking the meaning of words) and the raw score on the Matrix Reasoning Subtest (logical reasoning on abstract material) of the WAIS-III-R or IV.
- Processing speed was measured with raw scores on the Symbol subtest (crossing out as quickly as possible target symbols within a set of symbols over a 120 second time period) and percent of correct responses on the Coding subtest (writing down as quickly as possible the symbol corresponding to the digit following a digit-symbol code over a 120 second period) of the WAIS-III-R or IV.
- Verbal learning and memory were evaluated with the California Verbal Learning Test (CVLT)[47]. The CVLT is a 5 trial shopping-list learning test with immediate and delayed recalls, both free and semantically cued. The list consists of 16 words: 4 items from 4 semantically distinct categories. The CVLT also includes a final recognition task. The CVLT structure is well suited not only to study consolidation deficits but also to test acquisition difficulties. We selected one outcome measure from the CVLT, which included total learning trials 1 to 5, free delayed recall and recognition.
- Working Memory was evaluated with the percent of correct responses of the WAIS-III-R or IV Digit Span subtest in which the patients had to repeat a series of digits in correct and reverse order.
- Executive functions were assessed by 3 tests. Firstly, the Trail Making Test (TMT) [48] which also evaluates processing speed [49–51], and consists of 2 parts (A and B) that must be performed as quickly and accurately as possible. TMT-A requires subjects to draw lines sequentially to connect in ascending order, the 25 encircled numbers randomly distributed on a sheet of paper (i.e., 1–2–3–4, etc.). In TMT-B, the subject must alternate between numbers (1–13) and letters (A–L) while connecting them with lines (i.e., 1–A–2–B–3–C, etc.). TMT B-A completion time is usually used as an index of executive function since it reflects the ability for cognitive alternation and processing speed [52, 53]. Secondly, we used the Verbal Fluency Test which evaluates both verbal ability and executive control [54]. In this test, participants needed to retrieve words, which required them to access their mental lexicon. We used Semantic Fluency (the total number of animals named in 120 seconds) and Phonemic Fluency (the total number of words beginning with the letter “P” named in 120 seconds). Third, we used the Stroop Color-Word Interference Test (SCWT) [55]. This is believed to provide a measure of cognitive inhibition or the ability to inhibit an overlearned task (i.e., dominant response) in favor of an unusual one [56]. In this test, participants are required to read as many items as they can in 45 seconds from a card with 100 black-color words (W), a card with 100 colored XXXXs (C), and a card with 100 incongruent color words (WC). The outcome variables are the number of items completed for the word card (W: raw word score), the color card (C: raw color score), and the color–word card (CW: raw color–word score), respectively. We chose the Stroop Interference Score because it measures a cognitive form of inhibition known as interference control [57] so that a low score indicates poor inhibition performances. Interference scores are based on the following equation from the manual: CW raw score–[(W raw score × C raw score) / (W raw score + C raw score)].

### Statistical analyses

Data are expressed as proportions and frequency for categorical variables or means and standard deviations for continuous variables. Normality was assessed with the Shapiro-Wilk test. It has been suggested by the literature that age may be a major confounding factor of the association between adherence and neuropsychological functioning as it influences both dimensions. In order to investigate the specific effect of age, the 353 patients included were split into 2 age-groups based on the second tertile as 16–46 and 47–71 years. So as to verify that the 2 age-groups of patients (*16–46 and 47–71 years*) were comparable in terms of clinical, socio-demographic and neuropsychological characteristics, univariate analyses were performed. For continuous variables, the comparisons were made with Student tests or Mann-Whitney tests depending on the distribution of the variables while categorical variables were compared across the 2 groups using Chi-square tests. Univariate logistic regression analyses with adherence status according to the MARS score as the dependent variable (categorized as high or low adherent) were performed in both young and old bipolar patients groups separately in order to select the relevant clinical and neuropsychological predictors of nonadherence in both age’s groups. The selection of the predicting variables included in the regression models was based on a threshold of *p<0*.*20* in the univariate analysis. Then, stepwise multiple logistic regression analyses were conducted in both the young and the old groups separately with adherence status according to the MARS score as the dependent variable (categorized as high or low adherent) and the age specific predictors highlighted in each group as the independent variables. As we included neuropsychological raw scores in the model, we also included usual confounding variables in neuropsychological analysis such as education level, age and gender. Multicollinearity was examined by evaluating the Variance Inflation Factors (VIF) of the selected predictors when running the models in the 2 groups of patients. No multicollinearity issues were identified (with all VIF values <2). The threshold for statistical significance was defined to *p<0*.*05*. Data were analyzed using the Statistical Package of the Social Sciences (SPSS), version 20 (IBM Corporation, Armonk, NY, USA).

## Results

A total of 353 euthymic patients with BD from the FACE-BD cohort were enrolled in this study. The clinical, socio-demographic, and other characteristics of the patients are provided in Table 1. Most participants were females (61.8%) and the mean age of participants was 40.7 (SD = 12.7). The majority of the patients had obtained a graduate level diploma (37.1%). Patients were diagnosed with BD I (57.8%), with BD II (31.7%), or with BD NOS (10.5%).

**Table 1:** Socio-demographic and clinical description of the sample by age groups.

	Whole Sample (N = 353)	Age category		Statistics	p-value
		Young (N = 241)	Old (N = 112)		
*Sociodemographic characteristics*					
Age *Mean (SD)*	40.67 (12.66)	33.77 (8.07)	55.54 (6.39)	-	-
Sex (Male/Female) *N (%)*	135/218 (38.2/61.8)	86/155 (35.7/64.3)	49/63 (43.8/56.2)	Chi-square	*0*.*147*
Education category *N (%)*					
High school diploma incomplete	51 (14.4)	34 (14.1)	17 (15.2)	Chi-square	0.824
High school diploma obtained	51 (14.4)	37 (15.4)	14 (12.5)		
Bachelor incomplete or obtained	120 (34.0)	79 (32.8)	41 (36.6)		
Graduate level diploma obtained	132 (37.1)	91 (37.8)	40 (35.7)		
*Clinical variables*					
**Bipolar Disorder subtype**					
BD I *N (%)*	204 (57.8)	153 (63.5)	51 (45.5)	Chi-square	**0.005**
BD II *N (%)*	112 (31.7)	68 (28.2)	44 (39.3)		
BD Nos *N (%)*	37 (10.5)	20 (8.3)	17 (15.2)		
Adherence group (High /Low) *N (%)*	186/167 (52.7/47.3)	119/122 (49.4/50.6)	67/45 (59.8/40.2)	Chi-square	*0*.*067*
**Age at onset** *Mean (SD)*	24.4 (9.1)	21.54 (6.10)	30.52 (11.40)	*U* Mann-Whitney	**<0.001**
**Illness duration (years)** *Mean (SD)*	16.9 (11.4)	12.83 (8.08)	26.10 (12.47)	*U* Mann-Whitney	**<0.001**
Number of hospitalizations *Mean (SD)*	2.9 (2.7)	2.78 (2.72)	3.03 (2.79)	*U* Mann-Whitney	0.448
**Lifetime psychotic symptoms** *N (%)*	155 (53.4)	123 (60.0)	32 (37.6)	Chi-square	**0.001**
MADRS score *Mean (SD)*	4.1 (3.4)	4.14 (3.38)	3.95 (3.49)	*U* Mann-Whitney	0.514
YMRS score *Mean (SD)*	1 (1.6)	0.95 (1.59)	1.04 (1.53)	*U* Mann-Whitney	0.384
PRISE-M score *Mean (SD)*	10.3 (8.1)	10.71 (8.04)	9.55 (8.17)	*U* Mann-Whitney	*0*.*123*
STAI Y-A score *Mean (SD)*	36.3 (12.8)	36.75 (12.70)	35.23 (13.09)	*U* Mann-Whitney	*0*.*156*
*Comorbidities*					
**Lifetime Anxiety disorder** *N (%)*	76 (23.0)	61 (26.5)	15 (14.9)	Chi-square	**0.020**
**Lifetime Substance Use disorder** *N (%)*	87 (26.1)	68 (29.7)	19 (18.3)	Chi-square	**0.028**
Lifetime Smoking *N (%)*	174 (54.9)	124 (56.6)	50 (51.0)	Chi-square	0.354
*Treatment*					
Number of Medication *Mean (SD)*	1.8 (0.8)	1.76 (0.78)	1.82 (0.81)	*U* Mann-Whitney	0.565
Lithium *N (%)*	118 (33.4)	77 (32.0)	41 (36.6)	Chi-square	0.388
Anticonvulsants *N (%)*	187 (53)	129 (53.5)	58 (51.8)	Chi-square	0.760
**Antidepressants** *N (%)*	149 (42.2)	92 (38.2)	57 (50.9)	Chi-square	**0.024**
Typical Antipsychotics *N (%)*	26 (7.4)	18 (7.5)	8 (7.1)	Chi-square	0.913
Atypical Antipsychotics *N (%)*	136 (38.5)	101 (41.9)	35 (31.2)	Chi-square	*0*.*055*
*Neuropsychological variables Mean (SD)*					
**WAIS Symbols raw score**	32.93 (8.05)	34.80 (7.78)	28.83 (7.07)	*U* Mann-Whitney	**<0.001**
**WAIS Coding Percent correct**	50.10 (11.77)	52.94 (11.01)	43.83 (10.97)	*U* Mann-Whitney	**<0.001**
**Trail Making Test B—A Time (s)**	47.32 (36.95)	41.82 (27.75)	59.17 (49.56)	*U* Mann-Whitney	**0.001**
**CVLT List a Total 1–5 raw score**	56.60 (11.37)	58.11 (10.63)	53.39 (12.26)	*U* Mann-Whitney	**0.003**
**CVLT Recognition raw score**	15.04 (1.39)	15.23 (1.12)	14.64 (1.78)	*U* Mann-Whitney	**0.002**
**CVLT Free Delayed Recall raw score**	12.27 (3.15)	12.76 (2.79)	11.22 (3.60)	*U* Mann-Whitney	**<0.001**
WAIS Forward Digit Span Percent correct	56.91 (12.19)	57.70 (12.44)	55.23 (11.53)	*U* Mann-Whitney	0.109
**WAIS Backward Digit Span Percent correct**	45.56 (14.07)	47.71 (14.44)	41.02 (12.12)	*U* Mann-Whitney	**<0.001**
Phonemic Verbal Fluency raw score	22.60 (6.51)	22.76 (6.28)	22.23 (7.01)	*U* Mann-Whitney	0.505
Semantic Verbal Fluency raw score	30.66 (7.28)	30.81 (7.39)	30.33 (7.07)	*U* Mann-Whitney	0.747
SCWT Interference score	1.07 (7.90)	1.19 (8.18)	0.83 (7.30)	*U* Mann-Whitney	0.687

Abbreviations: *CVLT*, *California Verbal Learning Test; MADRS*, *Montgomerry Asberg Depression Rating Scale; NOS*, *not otherwise specified; PRISE-M*, *Patient Rated Inventory of Side Effects; SCWT*, *Stroop Color and Word Test; STAI-Y-a*, *State Trait Anxiety Inventory Y form assessing State Anxiety; WAIS*, *Wechsler Adult Intelligence Scale; YMRS*, *Young Mania Rating Scale*.

The univariate comparisons across the young and old bipolar patients subgroups showed that the 2 groups were comparable on numerous characteristics such as the gender repartition, the education level, the adherence status according to the MARS score, lifetime hospitalizations, the presence of residual symptoms (MADRS and YMRS scores), the level of adverse effects (PRISE-M score) and trait anxiety (STAI-Ya score), lifetime smoking status, medications except for the use of antidepressants, performances at verbal fluencies, SCWT and forward digit span (see Table 1 for more details). But there were also few statistically significant differences between the 2 age-groups, especially regarding neuropsychological performances, with the young patients performing better than their elders (see Table 1 for more details).

Before performing multiple stepwise logistic regression analyses, univariate logistic regressions of all the potential clinical, socio-demographic and neuropsychological predictors on the adherence category (low vs high) were conducted separately in the 2 age groups. In the young patients group, age, illness duration, number of hospitalizations, adverse effects, depressive residual symptoms and state anxiety score, history of lifetime substance abuse, current lithium medication and the SCWT interference score were the most associated factors to low adherence (Table 2). Whereas in the older group, the predictors retained to explain the adherence category were the illness duration, the number of hospitalizations, the adverse effects score, the history of lifetime smoking, the use of typical antipsychotics, the phonemic verbal fluency raw score and the SCWT interference score (Table 2).

**Table 2:** Univariate logistic regressions to predict low adherence (versus high adherence) in young (*16–46 years)* and old (*47–71 years)* bipolar patients’ subgroups.

	Age Bipolar patients’ subgroups			
	Young (N = 241)		Old (N = 112)	
	OR	*p*	OR	*p*
*Sociodemographics and clinical variables*				
Age	0.958	*0*.*009*	1.011	*0*.*719*
Sex—Female	1.117	*0*.*680*	0.954	*0*.*903*
Education—High school diploma incomplete	0.832	*0*.*648*	0.625	*0*.*450*
Education—High school diploma obtained	0.638	*0*.*256*	2.000	*0*.*271*
Education—Bachelor incomplete or obtained	1.177	*0*.*598*	0.960	*0*.*928*
Education—Graduate level diploma obtained	*-*	*0*.*474*	-	*0*.*486*
Bipolar Disorder subtype—BD I	0.851	*0*.*735*	0.922	*0*.*886*
Bipolar Disorder subtype—BD II	0.771	*0*.*611*	0.989	*0*.*985*
Bipolar Disorder subtype—BD Nos	-	*0*.*869*	-	*0*.*982*
Age at onset	0.987	*0*.*548*	1.020	*0*.*256*
Illness duration	0.944	*0*.*003*	0.977	*0*.*175*
Number of hospitalizations	0.829	*0*.*002*	0.826	*0*.*027*
Lifetime psychotic symptoms	1.363	*0*.*279*	0.633	*0*.*324*
MADRS score	1.139	*0*.*001*	1.055	*0*.*336*
YMRS score	0.965	*0*.*660*	1.022	*0*.*860*
PRISE-M score	1.078	*<0*.*001*	1.110	*0*.*001*
STAI Y-A score	1.023	*0*.*032*	1.018	*0*.*231*
Lifetime Anxiety disorder	0.839	*0*.*556*	0.747	*0*.*605*
Lifetime Substance Use disorder	0.589	*0*.*071*	0.671	*0*.*435*
Lifetime Smoking	1.319	*0*.*311*	0.560	*0*.*163*
Number of Medications	0.844	*0*.*309*	1.189	*0*.*468*
Lithium	0.632	*0*.*100*	0.927	*0*.*850*
Anticonvulsants	0.917	*0*.*736*	1.288	*0*.*513*
Antidepressants	0.895	*0*.*677*	1.589	*0*.*233*
Typical Antipsychotics	0.765	*0*.*587*	2.667	*0*.*195*
Atypical Antipsychotics	1.303	*0*.*312*	0.580	*0*.*205*
*Neuropsychological variables*				
WAIS Symbols raw score	1.017	*0*.*308*	0.974	*0*.*350*
WAIS Coding Percent correct	1.007	*0*.*538*	1.002	*0*.*913*
Trail Making Test B—A (Time)	1.005	*0*.*350*	1.000	*0*.*914*
CVLT List a Total 1–5 raw score	1.003	*0*.*783*	0.995	*0*.*772*
CVLT Recognition raw score	0.893	*0*.*338*	1.021	*0*.*848*
CVLT Free Delayed Recall raw score	1.004	*0*.*921*	1.007	*0*.*896*
WAIS Forward Digit Span Percent correct	1.001	*0*.*907*	0.995	*0*.*767*
WAIS Backward Digit Span Percent correct	0.998	*0*.*862*	0.991	*0*.*589*
Phonemic Verbal Fluency raw score	1.003	*0*.*893*	1.040	*0*.*181*
Semantic Verbal Fluency raw score	1.021	*0*.*250*	0.993	*0*.*802*
SCWT Interference score	1.037	*0*.*030*	0.934	*0*.*020*

Abbreviations: *CVLT*, *California Verbal Learning Test; MADRS*, *Montgomerry Asberg Depression Rating Scale; PRISE-M*, *Patient Rated Inventory of Side Effects; SCWT*, *Stroop Color and Word Test; STAI-Y-a*, *State Trait Anxiety Inventory Y form assessing State Anxiety; WAIS*, *Wechsler Adult Intelligence Scale; YMRS*, *Young Mania Rating Scale*.

The previous selected age specific predictors were entered in 2 separate multiple stepwise logistic regression analyses to explain low adherence. Age, gender and education category were also included in the regressions models as potential confounding variables. Table 3 presents the initial and final steps of the analysis. In the younger patients, the analyses reveal that the most associated factors to low adherence were a smaller number of hospitalizations (*OR* = 0.846, *p* = 0.012), a shorter illness duration (*OR* = 0.937, *p* = 0.003) and higher adverse effects (*OR* = 1.082; *p* = 4.457e-4). In the oldest patients, the factors retained to explain adherence were again a smaller number of hospitalizations (*OR* = 0.727, *p* = 0.008) and higher adverse effects (*OR* = 1.124, *p* = 0.005) but also poor inhibition performances evaluated by the SCWT interference score (*OR* = 0.924, *p* = 0.030).

**Table 3:** Separate multiple logistic regressions to predict low adherence (versus high adherence) in young (*16–46 years)* and old (*47–71 years*) bipolar patients’ subgroups before and after backward selection procedures.

Factors	β	*p*	Adjusted Odds Ratio	95% Confidence Interval
***Young (n = 241)***				
*Step 1*^a^				
Age	-0.022	*0*.*434*	0.979	0.927–1.033
Sex	-0.200	*0*.*585*	0.819	0.399–1.679
Education—High school diploma incomplete	-0.726	*0*.*168*	0.484	0.172–1.358
Education—High school diploma obtained	-0.875	*0*.*102*	0.417	0.146–1.188
Education—Bachelor incomplete or obtained	0.048	*0*.*905*	1.049	0.475–2.315
Education—Graduate level diploma obtained	-	*0*.*198*	-	-
Number of Hospitalizations	-0.185	*0*.*011*	0.831	0.720–0.959
Illness Duration	-0.043	*0*.*149*	0.958	0.903–1.016
PRISE-M score	0.067	*0*.*022*	1.069	1.010–1.132
Lithium medication	0.408	*0*.*252*	1.504	0.748–3.022
Lifetime Substance Use disorder	0.314	*0*.*412*	1.369	0.647–2.895
MADRS score	0.035	*0*.*569*	1.036	0.917–1.170
STAI Y-A score	0.014	*0*.*385*	1.014	0.982–1.047
SCWT Interference score	0.032	*0*.*120*	1.032	0.992–1.074
*Final Step*				
**Number of Hospitalizations**	-0.168	*0*.*012*	0.846	0.742–0.964
**Illness Duration**	-0.065	*0*.*003*	0.937	0.899–0.979
**PRISE-M score**	0.079	*<0*.*001*	1.082	1.035–1.131
***Old (n = 112)***				
*Step 1*^a^				
Age	0.005	*0*.*922*	1.005	0.909–1.112
Sex	-0.073	*0*.*907*	0.930	0.276–3.138
Education—High school diploma incomplete	0.435	*0*.*664*	1.546	0.217–11.012
Education—High school diploma obtained	0.688	*0*.*475*	1.989	0.302–13.117
Education—Bachelor incomplete or obtained	0.614	*0*.*385*	1.847	0.436–7.375
Education—Graduate level diploma obtained	-	*0*.*826*	-	-
Number of Hospitalizations	-0.257	*0*.*057*	0.773	0.594–1.007
Illness Duration	-0.049	*0*.*065*	0.952	0.903–1.003
PRISE-M score	0.143	*0*.*003*	1.153	1.049–1.268
Typical Antipsychotics	-0.704	*0*.*581*	0.495	0.041–6.041
Lifetime Smoking	1.059	*0*.*083*	2.884	0.869–9.568
Semantic Verbal Fluency Raw score	0.074	*0*.*137*	1.076	0.977–1.186
SCWT Interference score	-0.086	*0*.*044*	0.917	0.843–0.998
*Final Step*				
**Number of Hospitalizations**	-0.319	*0*.*008*	0.727	0.574–0.921
**PRISE-M score**	0.117	*0*.*005*	1.124	1.035–1.220
**SCWT Interference score**	-0.079	*0*.*030*	0.924	0.861–0.992

^a^The variables included in steps 1 are the variables with a p<0.20 in the univariate regression analyses (see Table 2).

Abbreviations: *MADRS*, *Montgomerry Asberg Depression Rating Scale; PRISE-M*, *Patient Rated Inventory of Side Effects; STAI-Y-a*, *State Trait Anxiety Inventory Y form assessing State Anxiety; SCWT*, *Stroop Color and Word Test*.

Antidepressant medication was not equally distributed within our sample which could influence medication adherence due to the side effects or potential neuroprotective effects of antidepressants. Therefore, the use of antidepressants could constitute a hidden confounding effect and the effect of antidepressant medication has been tested and the variable was forced in both backward logistic regression models. Of note, our main result was unchanged as inhibition performances were still a significant predictor of low adherence only in the older patients (OR = 0.923 [0.855–0.998], p = 0.044).

## Discussion

This is the first cross-sectional study investigating the link between neurocognition and low treatment adherence in BD with a particular focus on the effect of age. Interestingly, our result suggests that the association between adherence and executive functioning varies as a function of age. More precisely, we demonstrated that in older euthymic bipolar patients, poor inhibition performances predict low adherence while no significant effect was found in younger patients. In addition to this new finding, our study replicates the well-studied effect of the amount of adverse effects on adherence [5, 6] that experiencing a high level of adverse effects is a risk factor for low adherence but we proved that this effect has to be considered whatever the age of the patient. We also demonstrate that a small number of lifetime hospitalizations predicts low-adherence regardless of age. It suggests that greater number of hospitalizations can lead to better adherence which supports the hypothesis of a learning effect from these episodes and is consistent with previous observations indicating that having experienced fewer episodes is a risk factor for low-adherence in bipolar disorder patients [11]. Besides, this learning effect is also in line with our last result indicating that a short illness duration constitutes a risk factor for low-adherence but in young bipolar patients only.

Our main finding is consistent with several studies that demonstrated a decline in executive functioning with aging [23, 58], and another study which showed an age by disease interaction, with older patients with BD performing most poorly [59]. Moreover, our results are in accordance with some previous studies focusing on the relationship between adherence and neurocognition. Martinez-Aran et al investigated whether low treatment adherence is associated with cognitive impairment in 103 euthymic patients with BD [19]. They showed that patients with low adherence have significantly poorer performance on several cognitive functions. First, they were more impaired in the verbal learning and cued short recall tasks of CVLT than the other groups (i.e., high compliance and control), and furthermore, the Stroop Interference Score was lower in low adherent patients compared to controls. Finally, they demonstrated that TMT-B, which can also be considered as a measure of executive functioning, was significantly lower compared with both groups. However, after controlling for the confounding effect of several variables, only the TMT-B performance remained significant. These results also support an implication of executive processing in treatment adherence in BD even if the involved neuropsychological test was different. These differences might be explained by methodological differences and more restrictive inclusion criteria, that is, patients with less residual symptoms and without substance use disorder. Similarly, a study including 120 patients with BD with current depressed or mixed episode and cocaine dependence, found that baseline cognitive functioning measured by the Stroop Color–Word test and performance in simple visual attention tasks, assessed by the Stroop Word condition, was inversely associated with treatment adherence [20]. By contrast, another study [22] found no difference in neurocognitive performance according to adherence in a sample of 101 patients with BD, who were mostly euthymic, and 154 patients with a schizophrenia spectrum disorder. It is worth noting that these previous studies show conflicting results that might be explained by a limited sample size, the inclusion of non-euthymic patients vs. strictly euthymic patients or a confounding age effect.

Associations between unintentional adherence and neurocognitive function have been previously described in different medical conditions such as in hypertension, systematic lupus erythematosus, dementia and late-life depression [18, 60–62]. Even if the MARS was not originally designed to assess intentionality of adherence behavior, some authors have highlighted that the 2 first items of the MARS are an appropriate measure of unintentional non-adherence (i.e. “Do you ever forget to take your medication?”, “Are you careless at times about taking your medicine?”) while the others 8 items evaluate intentional non-adherence [63]. Based on these results, from an exploratory approach, we created two dimensions from the MARS’ items: intentional (sum of items 3–8) and unintentional (sum of items 1 & 2). Interestingly, in the older bipolar patients, the SCWT interference score was positively correlated with only the unintentional dimension of non-adherence (r = 0.26, p = 0.006).

To the best of our knowledge, only one previous study that included patients with HIV infection examined the hypothesis of an age effect in the relationship between neurocognition and medication adherence [27]. A sample of 431 HIV-infected adults was divided into two groups: younger (age < 50 years) and older. In this study, neurocognitive impairment was associated with poorer medication adherence among older participants only. When cognitive subdomains were examined individually, executive functioning, motor functioning, and processing speed were most strongly related to adherence in this age group. These results are in accordance with our findings.

Thus, our results emphasize the relevance of considering age in the relationship between adherence and neuropsychological performances, and underline the importance of executive functioning in adherence behavior in older patients. In psychiatric disorders, our study is the first to show this interaction between age and executive processing when focusing on poor adherence to treatment.

Two different plausible explanations that can be concurrent and may influence each other could be hypothesized to account for the association between cognitive functioning and adherence. One could consider that low adherence might be a consequence of poor executive functioning in older patients. Prospective memory, which is the “memory for activities to be performed in the future” [64], involves executive functioning such as cognitive flexibility and planning, which is assumed by the recruitment of the prefrontal cortex during prospective memory tasks [65–67]. Interestingly, deficits in prospective memory have been related to medication nonadherence in different clinical populations [67]. Furthermore, prospective memory has been shown to be lower in patients with BD even in remission phases [68, 69] and to decline with aging in the general population [70, 71]. Age-related executive impairments are also postulated to be associated with a deterioration of the frontal lobe [72, 73], and in BD further evidence of abnormal prefrontal cortical activity in remitted patients during performance of the Stroop task has been shown [74–76]. Therefore, we can hypothesize that a decline of executive functioning could lead to impairment in prospective memory and therefore an increased risk for medication nonadherence in older patients with BD.

An alternative explanation could account for the link between adherence and cognition. This posits that low adherence in BD may be a cause of poor executive functioning in older patients. Low adherence to medication leads to more relapses and recurrences of disease [7, 8], which cause impairment in cognitive functioning [77, 78]. It is reasonable to assume that older patients with BD are those who have accumulated the highest number of episodes and therefore have a greater risk of showing cognitive impairment. However, in our study, we have statistically controlled the analysis for the number of hospitalizations and the duration of illness which allows us to conclude that these variables have an independent effect on adherence. Consequently, the first hypothesis seems to be more plausible in the context of our study.

When extrapolating to clinical practice, our results suggest that targeted cognitive remediation programs might be proposed in addition to classic psychoeducative programs to improve medication adherence. Together, these approaches may enhance executive functioning, especially inhibition skills, in remitted patients over 47 years old with BD. A recent meta-analysis indicated that cognitive remediation in BD seemed to have promising results, but further studies are needed to evaluate the efficacy of interventions combining cognitive remediation and biological treatments [79].

The current study has several limitations. First, the use of a cross-sectional design and the absence of a healthy control group to control for the effect of natural aging require further longitudinal studies investigating the course of cognitive functioning and treatment adherence in regard to age. We suggest that it is important to clarify how aging, adherence, and neuropsychological functioning interact in the context of BD because current studies do not allow us to determine the mechanisms behind this observed association. Second, another methodological issue of our work is the definition of our low and good adherent groups and the categorization of age. However, the cut-off used for the MARS, ≥8, is the one recommended in previous studies [40, 43] and also corresponds to the median score of our sample (median score = 8; Inter-quartile range [6–9]). In fact, two post-hoc analyses have been conducted either adjusting the cut-off score to 6 or categorizing adherence into 3 groups in order to prevent a possible categorization induced bias. The effect of the SCWT interference score was replicated when considering a cut-off score of 6 (OR = 0.910 [0.843–0.982], p = 0.016) as well as when using 3 categories of adherence levels (OR = 0.877 [0.777–0.990], p = 0.033). Concerning the categorization of age in our sample, patients aged under the second tertile have been categorized as young (16–46 years) whereas patients aged over the second tertile have been categorized as old (47–71 years). This arbitrary categorical approach provides the advantage of facilitating the interpretation of the regression results. Accordingly, Johnson et al [80] described a quadratic influence of age on treatment adherence with a decrease up to 41 years and an increase beyond 41, which advocates for a binary categorization of age when studying adherence in BD. Third, some selection bias could be highlighted and moderates the generalization of our findings, such as the constrained criteria for euthymia and recruitment in tertiary specialized centers. It can be assumed that euthymic patients with few residual symptoms, who are followed in a Center of Expertise for BD represent a smaller specific category of BD patients who are probably more adherent. Moreover, this sample of bipolar patients were highly educated with about 2/3 having post-secondary education. Another limit of our study concerns the use of the stepwise backward selection procedure, in our logistic regressions analyses, that is known to inflate the Type I error rates (i.e., probability of erroneously rejecting a true null-hypothesis) due to multiple testing issues [81]. To limit this bias, the initial analyses were re-conducted with the ENTER method (variables selected if relevant according to the literature or if p<0.10 in univariate analyses). These new analyses produced comparable results, indicating that inhibition performances are significant predictors of low adherence only in older bipolar patients.

Finally, our study has several strengths. We selected the largest sample in comparison with previous studies cited above. We used strict euthymia as inclusion criteria. The neurocognitive battery used a wide range of measures which encompassed cognitive function impaired in BD and selected tests according to the International Society for Bipolar Disorders-Battery for Assessment of Neurocognition (ISBD-BANC) [46]. In addition, our protocol took into account a large set of potentially confounding variables and the resulting models lead to the correct classification of 67.5% of the young patients and 71.7% of the old patients as low or high adherent.

### Conclusions

In conclusion, our data suggest that adherence is associated to executive functioning in older patients only. In bipolar disorder, the impact of cognitive functioning on adherence may depend on age. It highlights the necessity of considering age in further studies and creating age-adapted therapeutic interventions to improve medication adherence.
